# Tiny Beads, Big Problems: Water Bead Ingestions—A Case Series

**DOI:** 10.3390/children12081041

**Published:** 2025-08-08

**Authors:** Jennifer M. Schuh, Hannah M. Olson, Kathleen M. Leack, Karlo Kovacic, Caroline Maloney, Jose H. Salazar

**Affiliations:** 1 Division of Pediatric Surgery, Department of Surgery, Medical College of Wisconsin, Milwaukee, WI 53226, USA; jmschuh@mcw.edu (J.M.S.);; 2 Division of Pediatric Surgery, Department of Surgery, Children’s Wisconsin, Milwaukee, WI 53226, USA; 3 Department of Pediatric Gastroenterology, Hepatology and Nutrition, Children’s Wisconsin, Milwaukee, WI 53226, USA

**Keywords:** water beads, Orbeez, bowel obstruction, superabsorbent polymer, ingestion, injury, case series

## Abstract

**Background:** Water beads are estimated to have caused >8000 emergency department (ED) visits from 2017 to 2022. Lethality after ingestion has been reported. The literature to guide management is scarce. We aimed to characterize three notable cases of water bead ingestions. **Methods:** We report the patient’s presentations, diagnostic modalities, clinical courses, intervention(s) required, and outcomes of the three children who ingested water beads. **Results:** All patients (ages 8 months–36 months old) presented to the ED with concern for water bead ingestion. Two developed clinical small bowel obstruction and required laparotomy—both required repeat laparotomy after further expansion of previously missed beads. The third patient ingested dozens of water beads but did not develop an obstruction or require surgery. X-rays, cross-sectional imaging, and ultrasounds were employed with variable results. **Conclusions:** While variable bead expansion rates can necessitate multiple interventions in some children, older children may be less prone to obstructing after water bead ingestion. This case series demonstrates the variability in presentation, management, and outcome of an increasingly common pediatric surgical problem.

## 1. Introduction

Water beads are superabsorbent polymer beads designed to expand by water absorption. They are used as children’s toys, decorations, and in a variety of products, including water bead guns (which shoot water bead projectiles), diapers, and agricultural materials. National data suggests that the number of water bead-related emergency department (ED) visits among the pediatric population has increased rapidly in recent years, more than doubling from 2021 to 2022 [1]. Water bead injury can occur from ear canal or nasal insertion, projectile injury, chemical contact, aspiration, or ingestion [1,2,3,4,5,6]. When marketed as toys or decorations, the beads are often sold in a dehydrated form with thousands of units per package. Often multicolored, the beads may appear like candy, tempting young children to ingest them. Ingestions account for the majority of water bead-related ED visits and hospital admissions in the US, with most impacted children ≤ 5 years old [1].

Thus far, water bead ingestions have been characterized by case reports, small case series, and a literature review summarizing these reports [3,5,6,7]. The focus has been on severe presentations or outcomes [5,6,8,9], but there is also some conflicting data to suggest that water bead ingestions often do not require medical intervention [1,10]. Recently, the national electronic injury surveillance system (NEISS), which reports a nationally representative probability sample from EDs within the United States, has also been leveraged to provide data about water bead-related injuries [1]. However, the NEISS data is limited in case-specific details and outcomes, like readmission to the hospital or treatment specifics, such as the need for an operation. It remains unclear what the typical clinical course is for these patients, which imaging modalities are effective for diagnosis and determination of management strategy, and whether there are specific risk factors linked to worse outcomes. Furthermore, there are no specific management guidelines. There have not yet been reports juxtaposing the treatment details of children who require surgical intervention with those treated with medical management alone.

Three cases of water bead ingestions are described, characterizing presentation, treatment course, and outcomes. Two children requiring multiple laparotomies for bowel obstruction are contrasted with one child who ingested hundreds of beads without developing an obstruction. These findings are contextualized within a review of the current literature on water bead ingestion.

## 2. Materials and Methods

Case details are reported directly from the pediatric surgical treatment team and obtained through retrospective chart review.

### 2.1. Ethics and Informed Consent

The treatment institution does not require ethical approval for reporting case series. Written consent for publication was obtained from one set of parents. Telephone consent, verified and documented by two providers, was obtained from the others.

### 2.2. Reporting

This case series is reported in accord with the Consensus Surgical CAse REport Guidelines (Supplemental Table S1) [11].

## 3. Patient Information

### 3.1. Patient 1

**Figure 1:**
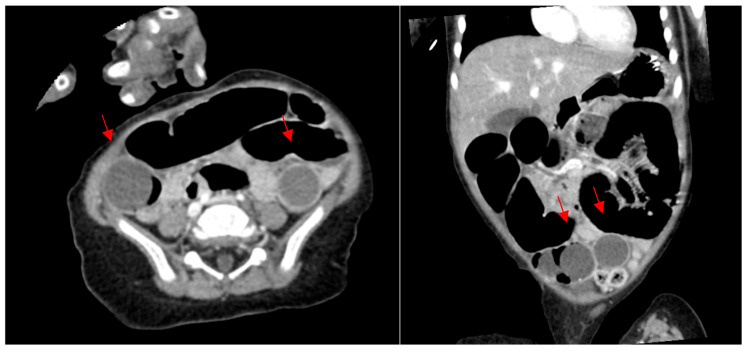
Preoperative computed tomography scan on a 14-month-old patient after water bead ingestion. Arrows indicate expanded beads.

A fourteen-month-old male with developmental delay presented to the ED of a children’s hospital with a one-day history of distension, decreased appetite, and vomiting; a single water bead was observed by parents in his emesis. Retrospectively, parents suspected water bead ingestion two days prior. On presentation, he was hemodynamically normal and afebrile. A computed tomography (CT) scan was obtained, which was consistent with a small bowel obstruction, and identified several spherical foreign bodies measuring 2–3 cm in diameter located in his pelvis (Figure 1). He was taken to the OR for an exploratory laparotomy (129 min total), during which eight water beads (diameters 0.5 cm–3 cm) were discovered. Five were causing complete obstruction approximately 15 cm proximal to the ileocecal valve, and were removed through a single longitudinal enterotomy, along with two smaller beads. Succus was evacuated through the enterotomy prior to transverse closure. One expanded bead was palpable in the sigmoid colon, advanced into the rectum, and removed transrectally. The entire small and large intestine was palpated for any remaining beads prior to closure.

**Figure 2:**
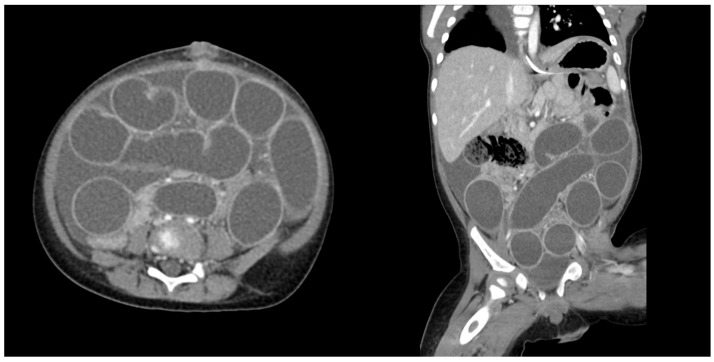
Postoperative computed tomography scan on a 14-month-old patient after water bead ingestion, prior to second look operation. Dilated loops of bowel visualized without distinct beads seen.

Postoperatively, the child developed an ileus, which was initially managed with bowel rest and nasogastric tube insertion. An X-ray obtained on postoperative day five demonstrated diffusely dilated bowel loops without an obstructive pattern or signs of a foreign body. On postoperative day seven, after further failure to improve, an X-ray demonstrated concern for an obstructive pattern. A CT was obtained, demonstrating two transition points with questionable visualization of rounded foreign bodies (Figure 2). He was taken back to the operating room for a repeat exploratory laparotomy (110 min duration). Two additional water beads were evident (3.1 and 3.5 cm)—one just proximal to his former enterotomy, and one in the mid-jejunum. They were expelled by reopening the prior enterotomy. Succus was again milked from the site, and the entire bowel was examined and palpated. A limited distal ileal resection and anastomosis were performed. After this operation, he recovered without complication (he did sustain an expected prolonged postoperative ileus), and was discharged from the hospital after a total 17-day length of stay.

### 3.2. Patient 2

Parents of an eight-month-old, 10.2 kg male with no pertinent medical or surgical history called their pediatrician with concerns of diminished alertness, associated with vomiting, decreased urine output, and failure to nurse for one day. Older siblings had spilled water beads on the floor near the child two days prior, and ingestion was suspected (father had removed two unexpanded beads from the child’s mouth at that time). Parents were advised to bring the child in for assessment, and they presented to a local ED. On presentation, the child was hemodynamically normal with a temperature of 37.8 Celsius. X-ray depicted two ovoid noncalcified soft tissue densities, one overlying the stomach (1.7 cm) and another within the right mid abdomen (2.75 cm); a nonspecific bowel gas pattern was read. He was transferred to a tertiary referral pediatric hospital promptly, where his abdominal examination was soft, non-distended, and non-tender. He was admitted to the pediatric surgical service for observation with serial X-rays and was permitted ad lib feeds.

**Figure 3:**
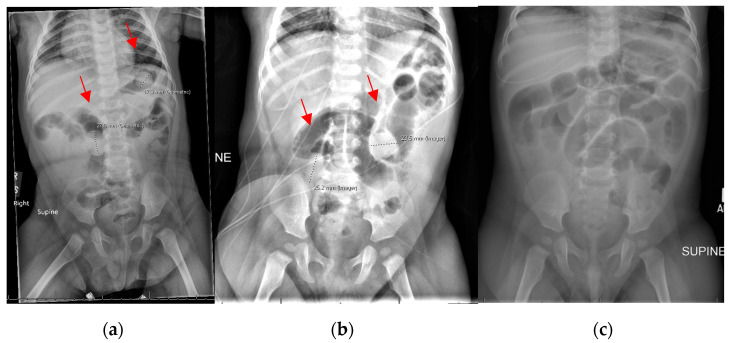
Serial abdominal X-rays obtained in an eight-month-old patient on hospital day one (**a**,**b**) and two (**c**) after admission for water bead ingestion. Arrows indicate expanded beads.

**Figure 4:**
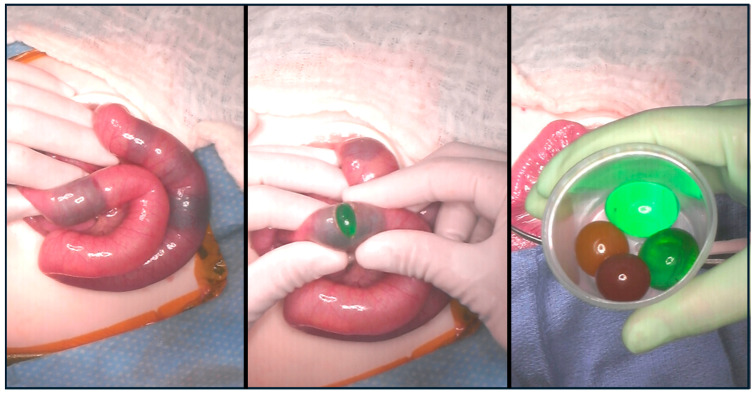
Intraoperative findings in an obstructed eight-month-old patient after water bead ingestion.

Serial imaging demonstrated increasing dilation of the proximal small bowel with a clinical increase in lethargy and poor oral intake (Figure 3); no more than two beads were visualized on any preoperative X-ray. On hospital day one, he was taken to the OR for diagnostic laparoscopy, which was converted to exploratory laparotomy (105 min duration), and four expanded water beads were removed from his distal ileum through an enterotomy (Figure 4).

**Figure 5:**
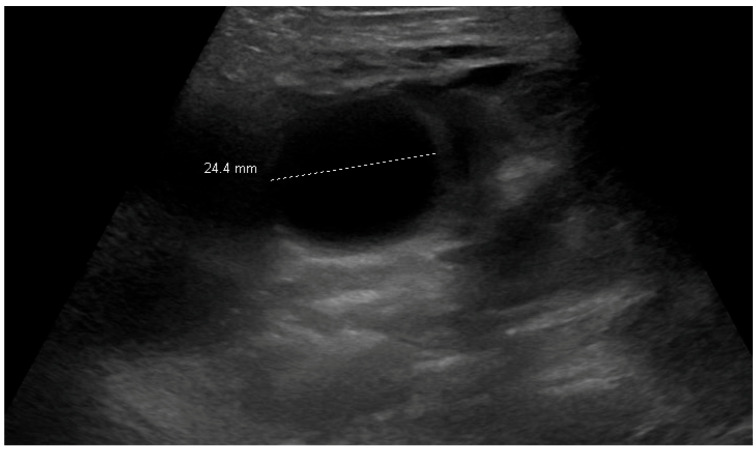
Ultrasound image obtained on postoperative day 4, depicting a rounded foreign body within the intestine.

After the operation, he did not tolerate a diet and had a few episodes of bilious emesis. At this time, his X-ray was unrevealing. However, as he was not clinically progressing, an abdominal ultrasound was obtained on postoperative day four, which depicted a rounded foreign body within the small intestine (2.7 cm, Figure 5). He was taken back to the OR and underwent a second exploratory laparotomy with the removal of the single missed additional foreign body (96 min duration); a segment of ileum was resected, and anastomosis was performed. An intraoperative esophagogastroduodenoscopy was performed to examine the proximal small bowel for any additional foreign bodies. After this operation, he recovered appropriately and was discharged (nine-day total length of stay).

### 3.3. Patient 3

**Figure 6:**
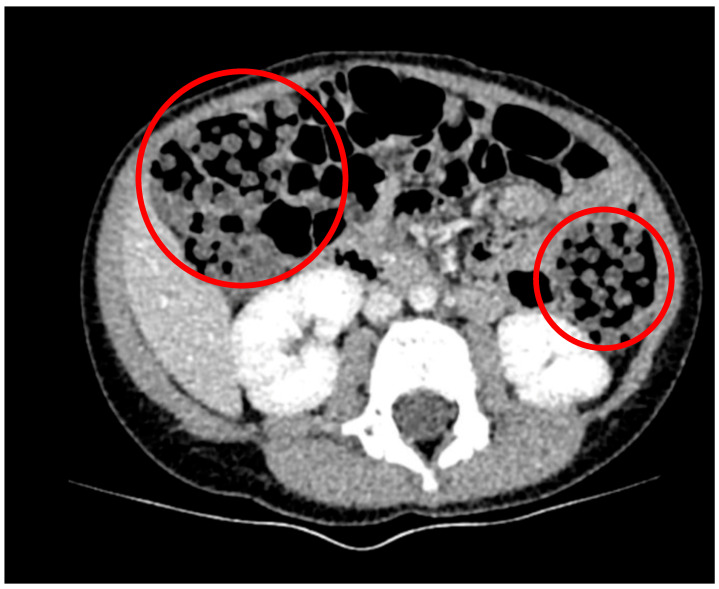
Computed tomography scan on a 36-month-old patient after water bead ingestion, managed nonoperatively. Circles indicate ingested beads.

A 36-month-old, 13.5 kg female presented to a local emergency room after one day of abdominal pain, vomiting, and defecation of numerous water beads. The mother reported finding water beads on the bed next to the patient on the night preceding presentation. She was afebrile and hemodynamically normal at the referring institution, and an X-ray revealed innumerable foreign bodies overlying her intestine without signs of obstruction; she was transferred to a tertiary referral children’s hospital. A CT was obtained, which also demonstrated innumerable foreign bodies without obstruction, approximately 6 mm in diameter (Figure 6). She was admitted with a clear liquid diet and received a mineral oil enema without subsequent defecation. The following day, she was advanced to a regular diet, managed with senna and erythromycin twice daily, and continued to pass diapers full of water beads. She was discharged on hospital day two without complication [12].

**Figure 7:**
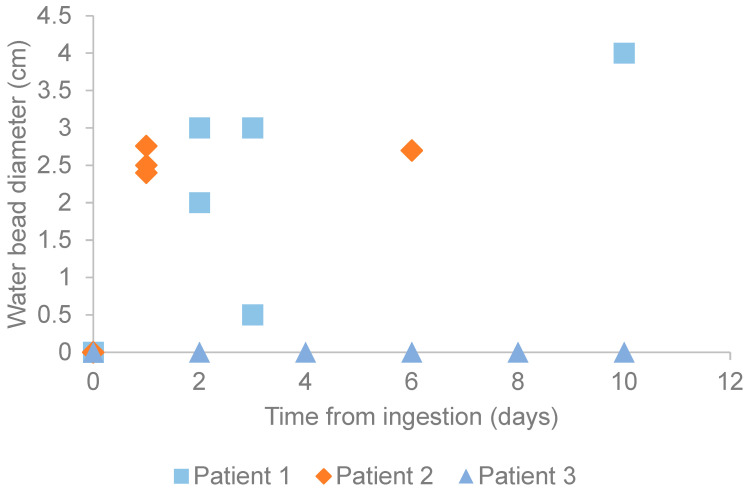
Time elapsed since ingestion compared to the diameter of the measured water bead (on imaging or in the operating room).

The ingested bead size for each patient relative to ingestion time is presented in Figure 7, underscoring the variability of expansion depending on bead chemical composition and patient physiologic parameters.

## 4. Discussion

This case series is the first direct comparison of the detailed treatment course for patients requiring surgical intervention for water bead ingestion with one who did not, despite evidence of innumerable beads ingested on CT. Imaging studies were varied in their ability to detect the water beads during the treatment course, with X-rays successfully identifying some expanded water beads but failing to detect other expanded and non-expanded beads. Ultrasound successfully identified an obstructing bead in one patient, and CT was able to detect unexpanded beads, but only in great quantities. This case series highlights the variability of presentation these patients may exhibit. Two patients required multiple laparotomies after ingesting a relatively small number of water beads. Meanwhile, the patient who ingested hundreds of beads cleared the beads without surgical intervention.

The patients who developed small bowel obstructions in our series were eight and fourteen months old. A literature review conducted in 2021 reported the outcomes of 43 patients (from 25 reports) who developed small bowel obstruction after water bead ingestions, with ages ranging from 6 to 36 months [5]. Relatives suspected water bead ingestion in only 10/43 cases of the obstruction review, whereas our sample is comprised entirely of children with confirmed or suspected water bead ingestion [5]. The report similarly identified ultrasounds as potentially the most sensitive imaging modality, with rounded intraluminal objects visualized in 82% (28/34) of cases who went on to develop bowel obstruction, compared to 100% (1/1) in our cohort. However, the appropriate diagnosis was only made in 15/34 of the cases in the review, while it was made expediently in our obstructed patient. The literature review on water bead obstructions reported a zero percent sensitivity of X-rays (31/43 had X-rays, with no foreign bodies visualized), which contrasts with some, but not all, water beads being visualized on X-rays in our cohort [5]. The difference in both ultrasound and X-ray sensitivity in our cohort likely reflects the higher index of suspicion afforded both by relatives reporting ingestion of water beads and the recent, dramatically increased prevalence of water bead ingestion. Furthermore, there may be a benefit afforded by a pediatric radiologist with extensive ultrasound experience at a children’s hospital reading these studies [1]. In this review, 41/43 patients required surgical intervention, and like our cohort, three patients required repeat laparotomy due to further expansion of retained water beads causing a recurrent bowel obstruction after their initial operation [5].

Our three patients underscore that medical management may be appropriate in the most carefully selected patients. However, there is little evidence published on the optimal medical management of water bead ingestions. In the patient who underwent successful medical management despite ingesting hundreds of beads (Patient 3), erythromycin was employed to stimulate peristaltic activity in the stomach and small intestine. An alternate stimulant such as azithromycin may be another reasonable approach [13]. Senna was used to aid the peristalsis of the colon [14]. Prucalopride, a highly selective serotonin receptor agonist, stimulates contractility of intestinal smooth muscle from the distal esophagus to the colon, and may be another consideration for select patients [15]. Using Gastrographin (diatrizoate), which may serve both diagnostic and therapeutic purposes, has also been proposed as a modality to treat water bead ingestion [16,17]. Treatment with osmotic laxatives, by contrast, may pose a risk by drawing fluid into the intestine and further expanding water beads. Certain brands of beads have even been demonstrated to reach maximal expansive capacity in polyethylene glycol solution [17]. Further, caution should be applied with secretory laxatives, due to their potential propensity for stimulating small bowel secretions.

Differential outcomes depending on the setting of presentation are underscored by a study examining ingestions of a specific water bead brand—Orbeez^TM^ (Spin Master; Los Angeles, CA, USA)—reported to the Texas poison control center from 2011 to 2016. Among 110 total reports, 78% of ingestions were successfully managed outside of a healthcare facility, with no reported cases of bowel obstruction or operative interventions [10]. NEISS data similarly demonstrated a need for admission in just 15% of patients presenting to the ED under age 5 [1]. Our series offers the advantage of looking at water bead ingestion concerns through the eyes of a pediatric healthcare provider, who may encounter the concern via phone call, in clinic, or in an emergency setting. The importance of understanding the signs and symptoms of bowel obstruction in the pediatric population is critical when assessing the need for admission in these cases.

Although an insufficient sample to draw statistically significant conclusions, these three patients do highlight meaningful characteristics to consider when making clinical decisions in how to manage a patient with water-beading ingestion. Our data is limited in that the brand of water bead ingested is unknown; however, brand and bead composition may significantly alter the obstructive propensity of the water bead, as in vitro studies have demonstrated certain brands are less likely to obstruct [7,17]. If the bead type (standard versus jumbo) is known at the time of presentation, this information, along with age, can inform providers’ index of suspicion for current or imminent bowel obstruction. However, frequently, the brand and type of bead are unknown at presentation and may be unreliable if reported. Therefore, it may be of more clinical relevance to characterize cases of ingestion without focusing on the reported bead type.

The patient experiences characterized in this report provide initial insight into practice management strategies for water bead ingestion but highlight opportunities for improvement to avoid repeat laparotomy, with the associated repeat anesthetic and prolonged hospitalization. A high index of suspicion must be maintained intraoperatively to identify beads that may not be fully expanded at initial laparotomy. Even when a high index of suspicion is maintained, beads can still be missed. Additionally, older children with a larger diameter of bowel who are not displaying signs of bowel obstruction may be more appropriately managed medically. Intraoperative techniques, including lavage and upper endoscopy, may provide useful adjuncts to prevent the need for reoperation. Preoperative magnetic resonance imaging (MRI) may also provide more detailed imaging and potentially prevent the need for multiple operations. However, MRI efficacy for this clinical scenario depends on slice thickness and water bead brand, as many unexpanded water bead diameters are only 1–3 mm, and may be difficult to discern within succus [17]. In these three cases, a variety of imaging modalities (X-rays, CT scans, and a single ultrasound) were meaningfully utilized. These cases lay the foundation for a more comprehensive review of water bead ingestions, including multi-institutional collaboration, to expand the generalizability and significance of these findings and establish a treatment guideline to optimize solutions for these children and their families. Reporting of these cases may serve to increase provider and parental awareness of the particular risk water beads pose and facilitate increased opportunity for age-appropriate anticipatory guidance in the primary care setting. These data may further serve to inform public healthcare policy as well as legislation, especially in light of recent legislative scrutiny on water bead toys [18,19,20].

## 5. Conclusions

Variable expansion rates after water bead ingestion can necessitate repeat interventions. Three patients presenting after water bead ingestion are reported. Younger age and progressive symptoms represent critical risk factors for surgical intervention and should be carefully considered in all patients with concern for water bead ingestion. X-rays are unreliable for the detection of expanded or unexpanded intestinal beads but can visualize expanded beads in some cases; ultrasounds likewise may have a high positive predictive value.

## Data Availability

The original contributions presented in this study are included in the article/Supplementary Materials. Further inquiries can be directed to the corresponding authors.

## Supplementary Materials

The following supporting information can be downloaded at: https://www.mdpi.com/article/10.3390/children12081041/s1, Table S1: Consensus Surgical Case Report Guidelines Adherence.

## Abbreviations

The following abbreviations are used in this manuscript:

CT	Computed Tomography
ED	Emergency Department
MRI	Magnetic Resonance Imaging
NEISS	National Electronic Injury Surveillance System
